# Early Pupillometry Assessment in Traumatic Brain Injury Patients: A Retrospective Study

**DOI:** 10.3390/brainsci11121657

**Published:** 2021-12-20

**Authors:** Thomas Luz Teixeira, Lorenzo Peluso, Pierluigi Banco, Hassane Njimi, Layal Abi-Khalil, Mélanie Chanchay Pillajo, Sophie Schuind, Jacques Creteur, Pierre Bouzat, Fabio Silvio Taccone

**Affiliations:** 1 Department of Intensive Care, Hopital Erasme, Université Libre de Bruxelles (ULB), 1050 Brussels, Belgium; thomas.luz.teixeira@ulb.be (T.L.T.); lorenzo.peluso@erasme.ulb.ac.be (L.P.); hassane.njimi@ulb.be (H.N.); layal.abi-khalil@ulb.be (L.A.-K.); melanie.chanchay.pillajo@ulb.be (M.C.P.); jcreteur@ulb.ac.be (J.C.); 2 Department of Anesthesiology and Intensive Care, University of Grenobles, 38400 Grenobles, France; pbanco@chu-grenoble.fr (P.B.); PBouzat@chu-grenoble.fr (P.B.); 3 Department of Neurosurgery, Hopital Erasme, Université Libre de Bruxelles (ULB), 1070 Brussels, Belgium; sophie.schuind@erasme.ulb.ac.be; 4 Grenoble Institute Neurosciences, University of Grenoble Alpes, 38700 Grenoble, France

**Keywords:** pupillometry, traumatic brain injury, outcome, prediction

## Abstract

Background: The aim of this study was to evaluate whether the early assessment of neurological pupil index (NPi) values derived from automated pupillometry could predict neurological outcome after traumatic brain injury (TBI). Methods: Retrospective observational study including adult (>18 years) TBI patients admitted from January 2018 to December 2020, with available NPi on admission. Abnormal NPi was considered if <3. Unfavorable neurological outcome (UO) at hospital discharge was considered for a Glasgow Outcome Scale of 1–3. Results: 100 patients were included over the study period (median age 48 (34–69) years and median GCS on admission 11 (6–15)); 49 (49%) patients had UO. On admission, 20 (20%) patients had an abnormal NPi (NPi < 3); median worst (i.e., from both eyes) NPi was 4.2 (3.2–4.5). Median worst and mean NPi on admission were significantly lower in the UO group than others (3.9 (1.7–4.4) vs. 4.4 (3.7–4.6); *p* = 0.005–4.0 (2.6–4.5) vs. 4.5 (3.9–4.7); *p* = 0.002, respectively). The ROC curve for the worst and mean NPi showed a moderate accuracy to predict UO (AUC 0.66 (0.56–0.77); *p* = 0.005 and 0.68 (0.57–0.78); *p* = 0.002). However, in a generalized linear model, the prognostic role of NPi on admission was limited. Conclusions: Low NPi on admission has limited prognostic value in TBI.

## 1. Introduction

Traumatic brain injury (TBI), with its variety in cerebral consequences (i.e., mild, moderate or severe), is one of leading causes of global morbidity and death, primarily in European countries [1]. The initial injury is often associated with the occurrence of secondary injuries, such as tissue hypoxia, seizures, or cerebral edema, which light further complicate with the development of intracranial hypertension and cerebral herniation. In the absence of early detection and prompt therapeutic response, these secondary injuries can lead to death or severe disability. As such, specific non-invasive and invasive neuro-monitoring systems have been implemented in clinical practice for TBI patients [2].

The cornerstone of neuromonitoring in this setting remains neurological examination, which is often simplified using clinical scales, together with the initial assessment of brain injuries [3,4]. Moreover, the evaluation of pupillary function (i.e., pupillary size, symmetry and the pupillary light reflex, PLR) can give an estimation of brainstem function and/or impelling herniation (i.e., anisocoria, fixed mydriasis, lack of PLR) [5,6] and has been introduced into extensive prognostic calculators to predict 6-month mortality and neurological outcome in adult patients with moderate to severe TBI on admission [7].

However, manual evaluation of pupil size and PLR using a pen torch has significant inaccuracies [8]; as such, the introduction of automated infrared pupillometers into clinical practice has improved the accuracy of pupillary assessment [9] and provide more reliable and quantitative data on pupil function at the bedside [10]. Moreover, one of these pupillometers can also provide the neurological pupil index (NPi), which is derived via a patented algorithm based on pupil size, constriction rate and velocity to light, latency of constriction and dilation velocity [11]. An abnormal NPi (i.e., <2 or 3) has been associated with poor outcome in patients suffering from post-anoxic brain injury or treated with veno-arterial extra-corporeal membrane oxygenation [12,13].

In a selected cohort of severe TBI patients with predominantly focal brain injury, NPi concomitantly decreased with episodes of elevated intracranial pressure (ICP); also, sustained abnormal NPi values were associated with worse outcome [14]. In another small cohort of blunt TBI, normal NPi on admission was observed in all patients who did not subsequently require neurosurgical interventions [15]. Nevertheless, there are limited data describing the prognostic role or a therapeutic predictive role of NPi and pupillometry in TBI patients.

The aim of this study was therefore to assess the role of early NPi assessment in TBI patients requiring on admission to the intensive care unit (ICU). Our hypothesis was that low NPi values could predict a poor long-term neurological outcome in this patients’ population. Also, the association of NPi and the intensity of brain-specific interventions was evaluated.

## 2. Materials and Methods

### 2.1. Study Population

A retrospective analysis was performed of all adult (>18 years of age) TBI patients admitted to the ICU of Erasme Hospital, Brussels, Belgium, between 10 January 2018 and 30 December 2020. Eligible patients were those having a NPi assessment on hospital admission, which was recorded into the patient data management system (PDMS, Picis Critical Care Manager, Picis Inc., Wakefield, MI, USA), as part of routine care. Exclusion criteria were the absence or incomplete NPi data as well as any ocular damage that could affect the pupillary examination (i.e., direct ocular trauma, cataract, blindness, glaucoma, previous eye surgery, severe periorbital edema). The study was approved by the ethical committee of the Erasme Hospital (Comité d’Ethique Hospitalo-Facultaire Erasme—ULB; P2021/035), which waived the need of informed consent given the retrospective observational design of the study.

### 2.2. Patients’ Management

The management of TBI patients followed general international recommendations [16]; care of these patients was under the responsibility of the senior ICU physicians, who discussed all cases into a multidisciplinary team including experienced neurosurgeons, neuro-radiologists and neurologists, according to patients’ severity. In severe TBI patients, sedation and mechanical ventilation was used, aiming to keep PaO_2_ and PaCO_2_ between 90–100 mmHg and 35–42 mmHg, respectively. Also, ICP monitoring was initiated and ICP kept below 20 mmHg, using a protocolized approach including osmotic therapy, sedation, moderate hyperventilation and increased cerebral perfusion pressure (CPP), from baseline values (i.e., 60–70 mmHg) to higher targets (i.e., 80–90 mmHg). In case of intracranial hypertension refractory to such interventions, extracranial ventricular drainage (EVD) was considered first, followed by “salvage” therapies, which included barbiturate coma, hypothermia or decompressive craniectomy, according to the characteristics of the brain injury. Metabolic control included the maintenance of normoglycemia (i.e., blood glucose between 110 and 150 mg/dL with the use of a continuous insulin infusion), normothermia (i.e., core body temperature < 37.5 °C) and early institution of enteral nutrition.

### 2.3. Automated Pupillometry

The assessment of pupillary function and PLR was performed using an automated pupillometer, the NeurOptics NPi-200 instrument (Neuroptics, Irvine, CA, USA), which uses an infrared camera that integrates a calibrated light stimulation of fixed intensity (1000 Lux) and duration (3.2 s) to provide rapid measurement (0.05 mm limit) of pupil size and quantitative PLR (i.e., the difference between baseline and post-stimulation pupil size, expressed as % of constriction from the baseline value), constriction velocity and latency. The measurement is completed in less than 30 s for each eye and a minimum duration of one minute was allowed between appraisals of the two pupils to obtain full recovery of baseline pupil diameter after light stimulation. The NPi was defined as normal (3–5) or abnormal (<3), as suggested by measurements performed in healthy subjects [11,17]. Also, the proportion of patients with a NPi < 2 was collected [12]. All pupillary evaluations were performed in complete darkness on hospital admission after the initial resuscitation maneuvers.

### 2.4. Data Collection

Together with the NPi, pupil size and constriction rate (CH) for both eyes on admission, we also collected data on demographics, comorbid diseases, mechanism of trauma, ICU and hospital length of stay, ICU and hospital mortality. The severity of disease on admission was estimated using the Sequential Organ Failure Assessment (SOFA) score [18]. TBI severity was estimated using the Glasgow Coma Scale (GCS) on admission [19] and the Marshall scale on the initial cerebral CT-scan [20].

The use of different therapies (i.e., mechanical ventilation, sedative, analgesic, vasopressor, inotrope, antiepileptic, barbituric and/or osmotic drugs), as well as different interventions (i.e., ICP monitoring, hypothermia, hypocapnia and decompressive craniotomy) was collected, together with relevant biochemical and physiological parameters. Neurological outcome at hospital discharge was assessed using the Glasgow Outcome Scale; favorable neurological outcome (FO) was considered as a GOS 4–5, while unfavorable outcome (UO) as GOS 1–3 [21]. The predicted outcome was calculated using the TBI-IMPACT Score in its extended format (core model ± CT ± Lab): unfavorable outcome and mortality were expressed as probability (i.e., percentages) [7]. The intensity of TBI management, in particular to control ICP, during the ICU stay was assessed using the Therapy Intensity Level (TIL)-Basic Score [22]; high intensity of care was defined as TIL of 4.

### 2.5. Study Outcomes

The primary outcome of the study was the prognostic role of the worst NPi value on the occurrence of UO. Secondary outcome included the prognostic role of the worst NPi on the occurrence of ICU mortality and the difference in NPi values across different TIL ranges and predicted UO and mortality according to the TBI-IMPACT database.

### 2.6. Statistical Analyses

Data were analyzed using R statistical software version 4.0.3 (R Foundation for Statistical Computing, Vienna, Austria), Prism (GraphPad Software Inc., San Diego, CA, USA) and IBM SPSS Statistics for Macintosh 27 (Armonk, NY, USA). Discrete variables were expressed as count (percentage) and continuous variables as median (25th to 75th percentiles). The Kolmogorov-Smirnov test was used, and histograms and normal-quantile plots were examined to verify the normality of distribution of continuous variables. Demographics, clinical, and pupillary patterns differences between groups (UO vs. FO; survivors vs. non-survivors) were assessed using the chi-square test or Fisher’s exact test for categorical variables and Mann–Whitney U-test or *t* Student test were used for continuous variables, as appropriate. Linear correlation between variables was assessed using Pearson’s or Spearman’s coefficients, accordingly. For multiple group comparisons, the Kruskal Wallis test, with Dunn-Bonferroni post hoc analysis, was used.

In the multivariable analyses, considering the limited number of events, the predictor variables that were highly collinear within and across outcome and the risk of overfitting, we used a generalized linear model via regularized regression with elastic net. Elastic net regression is controlled by two parameters, (1) α, which sets the degree of mixing between two extremes of regularized regression, and (2) λ, defining the strength of regularization [23]. Linearity of the continuous variables with respect to the logit of the dependent variable was assessed via the Box-Tidwell procedure. Presence of outliers were assessed by studentized residuals. The ability of NPi to predict UO or ICU mortality was tested with different receiving operating characteristics (ROC) curve, and the area under the curve (AUC) for each subgroup of patients was calculated. All tests are two tailed and the statistical significance was set at the 5% level.

## 3. Results

### 3.1. Study Population

During this study period, 136 TBI patients were admitted to the ICU; of those, 36 patients had incomplete or absent NPi assessment on admission, leaving 100 eligible patients for the final analyses (Supplemental Figure S1). Characteristics of the study population are shown in Table 1; median age was 48 (34–69) years and median GCS on admission was 11 (6–15). The overall ICU length of stay was 6 (3–17) days; ICU mortality occurred in 27 (27%) patients and UO at hospital discharge was observed in 49 (49%) patients.

### 3.2. NPi and Neurological Outcome

**Figure 1:**
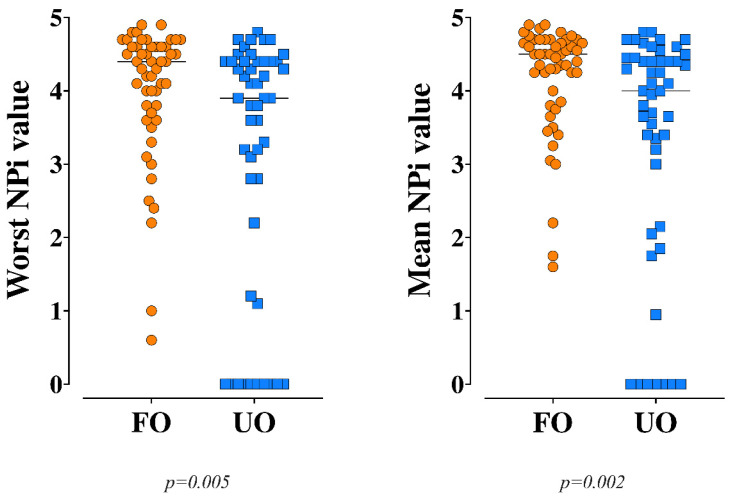
Worst and mean Neurological Pupil Index (NPi) on admission between patients with unfavorable (UO) and favorable (FO) neurological outcome. The Mann–Whitney U-test was used for this analysis.

Median worst NPi on admission was 4.2 (3.2–4.5); a total of 20 (20%) and 13 (13%) patients had a NPi < 3 and <2 on admission, respectively. Pupil size and constriction rate are reported in Table 1. Patient with UO were older and had significantly lower GCS and higher SOFA scores on admission. Also, patients with UO suffered more frequently from liver cirrhosis and arterial hypertension, had longer ICU length of stay and received a higher TIL when compared to those with FO (Table 1). Predicted UO using the TBI-IMPACT calculator was 64 (36–75)% and 37 (18–56)% (*p* = 0.006) in the UO and FO group, respectively. Median worst and mean NPi on admission were significantly lower in the UO group than the other (3.9 (1.7–4.4) vs. 4.4 (3.7–4.6); *p* = 0.005—4.0 (2.6–4.5) vs. 4.5 (3.9–4.7); *p* = 0.002, respectively—Figure 1).

Also, there were more patients with NPi <3 (14/49, 29% vs. 6/51, 12%; *p* = 0.046) and <2 (11/49, 22% vs. 2/51, 4%; *p* = 0.007) on admission in the UO than the FO group. The ROC curve for the worst and mean NPi showed a moderate accuracy to predict UO (AUC 0.66 (0.56–0.77); *p* = 0.005 and 0.68 (0.57–0.78); *p* = 0.002), which were similar to GCS on admission (AUC 0.70 [0.60–0.81]; *p* < 0.001 Table 2).

**Figure 2:**
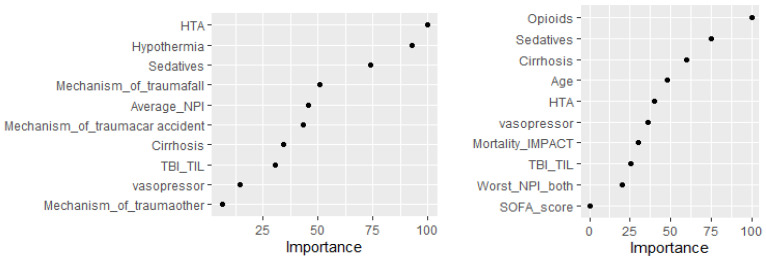
Contribution of selected variables to predict unfavourable neurological outcome (left Panel) or ICU mortality (right Panel). HTA = hypertension; NPi = neurological pupil index; TIL = Therapy Intensity Level; SOFA = Sequential Organ Failure Assessment.

In the regularized regression models with elastic net, mean NPi was associated with UO; however, its contribution was quite limited and less relevant than other variables, such as arterial hypertension, mechanism of trauma, use of sedatives or of hypothermia (Figure 2 and Table 3).

### 3.3. Secondary Outcomes

Median worst but not mean NPi on admission was significantly lower in non-survivors when compared to survivors; other differences between non-survivors and survivors are reported in Supplemental Table S1.

The proportion of patients with abnormal NPi on admission was similar between non-survivors and survivors. The ROC curve for the worst NPi showed a moderate accuracy to predict ICU mortality (AUC 0.66 (0.54–0.78); *p* = 0.013), which was similar to GCS on admission. In the regularized regression models with elastic net, worst NPi was associated with ICU mortality, however its contribution was very limited (Supplemental Table S2 and Figure 2).

**Figure 3:**
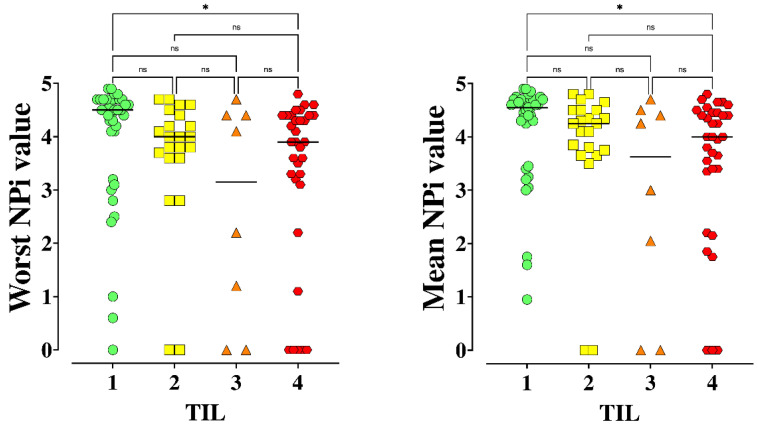
Worst and mean Neurological Pupil Index (NPi) values across different Therapeutic Intensity Level (TIL) ranges. * *p* < 0.05 for post hoc analysis among groups. NS = not statistically significant. The Kruskal Wallis test, with Dunn-Bonferroni post hoc analysis, was used.

A statistically significant difference in the worst and mean NPi on admission was observed across different TIL values (Figure 3; *p* = 0.01).

**Figure 4:**
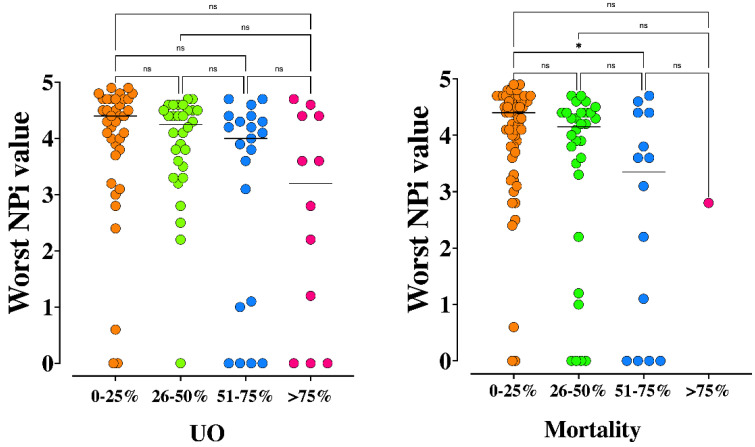
Worst Neurological Pupil Index (NPi) values across different predicted 6-month unfavorable neurological outcome (UO, *p* = 0.04) and mortality (*p* = 0.01) according to the TBI-IMPACT database. * *p* < 0.05 for post hoc analysis among groups. NS = not statistically significant. The Kruskal Wallis test, with Dunn-Bonferroni post hoc analysis, was used.

**Figure 5:**
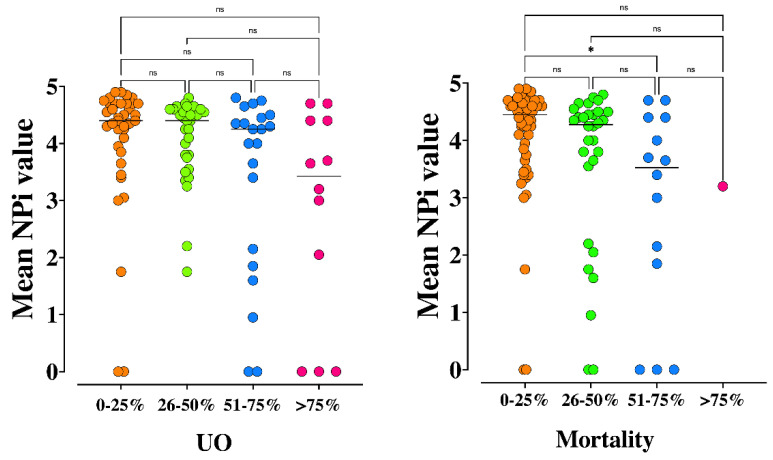
Mean Neurological Pupil Index (NPi) values across different predicted 6-month unfavorable neurological outcome (UO, *p* = 0.04) and mortality (*p* = 0.02) according to the TBI-IMPACT database. * *p* < 0.05 for post hoc analysis among groups. NS = not statistically significant. The Kruskal Wallis test, with Dunn-Bonferroni post hoc analysis, was used.

Differences of patients according to high intensity (TIL-4) or other TIL values are shown in Supplemental Tables S3 and S4. Also, worst and mean NPi on admission were significantly different when analyzed across different predicted UO and mortality rates according to the TBI-IMPACT Database (Figure 4 and Figure 5).

## 4. Discussion

In this study, early NPi on admission was abnormal in 20% of TBI patients. In particular, lower worst and mean NPI values were observed in patients with UO or non-survivors when compared to others. Also, lower NPi was observed in patients requiring high intensity of care for elevated ICP during the ICU stay. Nevertheless, the prognostic role of NPi was relatively limited in this setting.

Pupillary reactivity on admission has been largely used into predictive models of TBI patients [7]; TBI patients with GCS of 3 and bilateral fixed and dilated pupils have a significantly reduced likelihood of survival when compared to others [5], but many patients would have normal or sluggish pupil constriction to light and this finding would therefore have limited prognostic value in this setting. Moreover, PLR assessed with a penlight is highly inaccurate in brain injured patients and associated with a great inter-examiner variability [24]. In this setting, AP provides a more precise, reliable, reproducible and objective pupillary assessment [10,25] and can quantify the pupil function thorough the NPi. El Ahmadieh et al. proposed that AP may be useful as a screening tool in TBI patient, as they found that NPI < 3 is a predictor factor associated with the requirement of urgent neurosurgical intervention [15]. Worst median NPi values were also significantly lower in patients requiring surgery when compared to others (2.7 vs. 4.2; *p* ≤ 0.001). Patients with normal NPi on admission had a better neurological outcome at 3 months than others. Similarly, Park et al. reported that abnormal NPi values were strongly associated with lower GCS on admission in TBI patients [26]; however, NPi was only weakly correlated with ICP values.

In this study, NPi < 3.4 was the optimal cut-off to predict (i.e., sensitivity of 86% and specificity of 84%) neurological outcomes at 1 month, which was similar to our results. Jahns et al. also showed that abnormal NPi values were more frequent in patients with refractory intracranial hypertension and were associated with an unfavorable 6-month neurological outcome [14]. In this study, we included a more heterogeneous (i.e., mild to severe TBI) population and only patients requiring ICU admission; in this setting, NPi has a similar predictive value than GCS. Moreover, the predictive accuracy for the worst or mean NPi on admission to predict UO was quite limited. As such, future studies should specifically evaluate the role of NPi in severe TBI patients, in whom clinical examination is less reliable or altered by the administration of sedatives [24]; in those patients, adding a precise assessment of the pupillary function could increase the predictivity of the initial clinical assessment to identify patients at high risk of poor recovery. Moreover, we only assessed NPi on admission; in other studies, NPi assessment at 24 or 48 h after the anoxic injury [12] or repeated NPi assessment assessing specific trajectories [13] have shown to provide more prognostic information than initial NPi measurements. Finally, this is the first study which tried to adjust the association between NPi and outcome for potential confounders; this more accurate statistical approach was able to better quantify the contribution of NPi on outcome prediction than simple univariate associations. As such, although NPi on admission could be useful as a “triage” tool to identify “high-risk” TBI patients, the prognostic role of such variable needs to be further explored.

One of the validated predictive scores in moderate to severe TBI is the TBI-IMPACT score, with or without its extended model (i.e., adding cerebral CT-scan findings and laboratory values) [7]. We found an association of lower NPi values on admission with increasing predicted UO and mortality by this score. Nevertheless, we could not specifically calculate the additional role of NPi in this setting, as TBI-IMPACT calculation does not provide a specific value, such as prognostic scores, but only a probability of poor outcome. Also, the score has been validated in cohorts including thousands of TBI patients and it would be difficult to assess the interaction between this score and NPi in a limited and selected TBI population. As such, whether high TBI-IMPACT score and lower NPi values on admission would be more accurate than the score alone to predict outcome remains unknown from our findings.

Some studies have reported an association between reduced NPi values and increased ICP after TBI; in severe TBI patient, changes in ICP after administration of an osmotic therapy mirrored the increase in NPi values [14]. Similarly, in another study, NPi values < 3 were associated with high ICP and early NPi deterioration could also predict the rise in ICP [27]. In another study, recovery of NPi was observed within 2 h in TBI patients treated with osmotics, in particular if the pre-intervention NPi was below 3 [28]. In our study, we used the TIL score to assess the intensity of ICP-directed interventions; lower NPi values on admission were observed in those patients with the highest TIL during the ICU stay. This finding is interesting, as it might help to identify patients developing intracranial hypertension who will require the highest intensity of care (i.e., barbiturates, decompressive craniectomy or hypothermia). Whether low NPi could also predict the time to the requirement of these salvage therapy needs to be further clarified.

This study has several limitations to acknowledge. First, the small sample size, monocentric and retrospective design might introduce some significant selection biases and limit the generalizability of our findings. Second, we did not investigate all AP-derived variables but focused only on NPi, as this has been suggested as an important prognostic tool in this setting. Third, we did not record subsequent NPi values and no information on the prognostic role of NPi changes over time could be reported. Forth, many TBI patients can also suffer from polytrauma and another organ injury, such as chest or abdominal trauma, could have been critical and lead to death, independently from the initial cerebral injury, which would limit the prognostic value of NPi.

## 5. Conclusions

In this heterogeneous population of TBI patients, a lower NPi score on admission was observed in patients with poor neurological outcome at hospital discharge and high intensity of care for elevated ICP when compared to others. The overall prognostic contribution of NPi remained relatively limited and should be further evaluated in at-risk patients, such those with severe TBI.

## Figures and Tables

**Table 1:** Characteristics of the study population, according to neurological outcome at hospital discharge. Data are presented as count (%) or median (25th–75th percentiles); differences for categorical variables were assessed using the chi-square test (or the Fisher’s exact test when *n* < 10), while for continuous variables the Mann–Whitney U-test was used. UO = Unfavorable Outcome; FO = Favorable Outcome; NPI = Neurological Pupil Index; SOFA = Sequential Organ Failure Assessment; CT = Computed Tomography; CH = Change (percentage of constriction); COPD = Chronic Obstructive Pulmonary Disease; AHT = Arterial Hypertension; ECMO = Extracorporeal Membrane Oxygenation; ICP = Intracranial Pressure; ICU = Intensive Care Unit; IMPACT = International Mission for Prognosis and Analysis of Clinical Trials in TBI. * indicates fall from more than 3 m. Fall from smaller height are included into “Others”.

Variables	ALL (***n*** = 100)	UO (***n*** = 49)	FO (***n*** = 51)	*p*-Value
Age, years	48 (34–69)	55 (39–75)	45 (32–62)	0.04
Male Gender, *n* (%)	73 (73)	32 (65)	41 (80)	0.12
Mechanism of Trauma *n* (%)				0.02
Fall *	64 (64)	38 (78)	26 (51)	
Car accident	24 (24)	9 (18)	15 (29)	
Aggression	9 (9)	1 (2)	8 (16)	
Other	3 (3)	1 (2)	2 (4)	
Polytrauma, *n* (%)	38 (38)	17 (35)	21 (41)	0.54
Glasgow Coma Scale on admission	11 (6–15)	8 (4–13)	14 (8–15)	<0.001
SOFA Score on admission	6 (2–8)	9 (5–10)	3 (1–6)	<0.001
Marshall Score	5 (2–5)	5 (3–5)	4 (2–5)	0.35
Neurological Pupil Index				
Worst NPi on admission	4.2 (3.2–4.5)	3.9 (1.7–4.4)	4.4 (3.7–4.6)	0.005
Mean NPi on admission	4.3 (3.4–4.6)	4 (2.6–4.5)	4.5 (3.9–4.7)	0.002
NPi < 3, *n* (%)	20 (20)	14 (29)	6 (12)	0.046
NPi < 2, *n* (%)	13 (13)	11 (22)	2 (4)	0.007
Worst size (max) on admission, mm	3.1 (2.4–4.2)	2.9 (2.3–4.1)	3.2 (2.7–4.2)	0.39
Worst CH (min) on admission, %	16 (9–26)	12 (6–20)	18 (11–31)	0.008
Comorbidities				
COPD, *n* (%)	8 (8)	4 (8)	4 (8)	1
Chronic kidney disease, *n* (%)	7 (7)	6 (12)	1 (2)	0.06
Liver Cirrhosis, *n* (%)	5 (5)	5 (10)	0	0.02
Heart disease, *n* (%)	24 (24)	16 (33)	8 (16)	0.06
Immunosuppression, *n* (%)	1 (1)	1 (2)	0	0.49
Previous neurological disease, *n* (%)	19 (19)	13 (27)	6 (12)	0.08
Cancer, *n* (%)	5 (5)	3 (6)	2 (4)	0.67
Diabetes, *n* (%)	12 (12)	5 (10)	7 (14)	0.76
Arterial Hypertension, *n* (%)	25 (25)	19 (39)	6 (12)	0.002
Alcohol, n (%)	40 (40)	20 (41)	20 (39)	0.99
Smoking, *n* (%)	22 (22)	8 (16)	14 (28)	0.23
ICU Therapies				
Therapy Intensity Level (TIL-Basic)	2 (1–4)	4 (2–4)	1 (1–2)	<0.001
Sedative drugs, *n* (%)	68 (68)	45 (92)	23 (45)	<0.001
Analgesic drugs, *n* (%)	81 (81)	46 (94)	35 (69)	0.002
Vasopressor drugs, *n* (%)	56 (56)	38 (77)	18 (65)	<0.001
Inotropes drugs, *n* (%)	2 (2)	2 (4)		0.24
Mechanical ventilation, *n* (%)	69 (69)	44 (90)	25 (49)	<0.001
ECMO, *n* (%)	1 (1)	1 (2)		0.49
ICP Monitoring, *n* (%)	45 (45)	32 (65)	13 (26)	<0.001
Osmotic drugs, *n* (%)	46 (46)	32 (65)	14 (28)	<0.001
Decompressive craniectomy, *n* (%)	29 (29)	22 (45)	7 (14)	<0.001
Barbiturates, *n* (%)	17 (17)	14 (29)	3 (6)	0.003
Hypothermia, *n* (%)	6 (6)	6 (12)		0.012
Outcome Variables				
ICU stay, days	6 (3–17)	12 (4–23)	2 (3–7)	0.01
Hospital stay, days	16 (8–43)	22 (9–42)	15 (8–43)	0.61
ICU death, *n* (%)	27 (27)	27 (55.1)		<0.001
Hospital death, *n* (%)	29 (29)	29 (59.2)		<0.001
IMPACT Mortality, %	34 (15–50)	44 (24–57)	21 (12–37)	0.011
IMPACT Unfavourable Outcome, %	50 (24–71)	64 (36–75)	37 (18–56)	0.006

**Table 2:** Receiving Operating Characteristics (ROC) Curves to predict unfavourable neurological outcome at hospital discharge. NPi = Neurological Pupil Index; CT= Computed Tomography; CH = Change (percentage of constriction); GCS =Glasgow Coma Scale; IMPACT = International Mission for Prognosis and Analysis of Clinical Trials in TBI; TIL = Therapy Intensity Level.

Variables	AUC [IC 95%]	*p*-Value
Marshall score	0.57 (0.41–0.73)	0.37
TIL	0.63 (0.48–0.78)	0.11
IMPACT score	0.72 (0.59–0.85)	0.006
GCS on admission	0.70 (0.60–0.81)	<0.001
Worst NPi on admission	0.66 (0.58–0.77]	0.005
Mean NPi on admission	0.68 (0.57–0.78)	0.002
Worst Pupil Size on admission	0.45 (0.33–0.56)	0.37
Worst CH on admission	0.65 (0.545–0.76)	0.008

**Table 3:** Regularized regression models with elastic net. The fitted models showed an accuracy of 78% (CI 68–86%), a sensitivity 97%, a specificity 70%, a positive predictive value of 57% and a negative predictive value of 98% to predict unfavorable neurological outcome (UO). The fitted models showed an accuracy of 87% (CI 79–93%), a sensitivity 94%, a specificity 69%, a positive predictive value of 83% and a negative predictive value of 88% to predict ICU mortality. NPi = Neurological Pupil Index; SOFA = Sequential Organ Failure Assessment; TIL = Therapy Intensity Level. The parameter alpha has been selected according to the minimization of the partial likelihood deviance of the model; the lambda parameter was determined using grid search with 10-fold cross-validation and the optimal value was determined by minimizing the deviance of the model selected.

	UO	Mortality
	Coefficient	Coefficient
(Intercept)	−0.009	−2.62
SOFA score on admission	0.207	0.251
Age		0.767
Mean NPi	−0.627	
Worst NPi		−0.468
Arterial Hypertension	1.128	0.687
Liver Cirrhosis	0.524	0.898
Vasopressors	0.342	0.641
Sedatives	0.889	1.057
Opioids		1.328
Hypothermia	1.059	
TIL Score	0.486	0.528
Mechanism of trauma	0.602	

## Data Availability

Data are available on request to the corresponding author.

## Supplementary Materials

The following are available online at https://www.mdpi.com/article/10.3390/brainsci11121657/s1, Figure S1: Flow-chart of the study, Table S1: Characteristics of the study population, according to hospital mortality, Table S2: ROC Curves according to prediction of hospital mortality; Table S3: Characteristics of the study population, according to TIL-Basic, Table S4; ROC Curves according to the prediction of high TIL during ICU stay.
