# Supplementary material for: Early Pupillometry Assessment in Traumatic Brain Injury Patients: A Retrospective Study
Source: Brain Sci. 2021 Dec 20;11(12):1657. doi: 10.3390/brainsci11121657 (PMC8699519; doi:10.3390/brainsci11121657)
Supplement: Supplementary file 1 [file brainsci-11-01657-s001.zip › brainsci-1442526-supplementary.pdf]

Supplemental Figure S1. Flow-chart of the study.

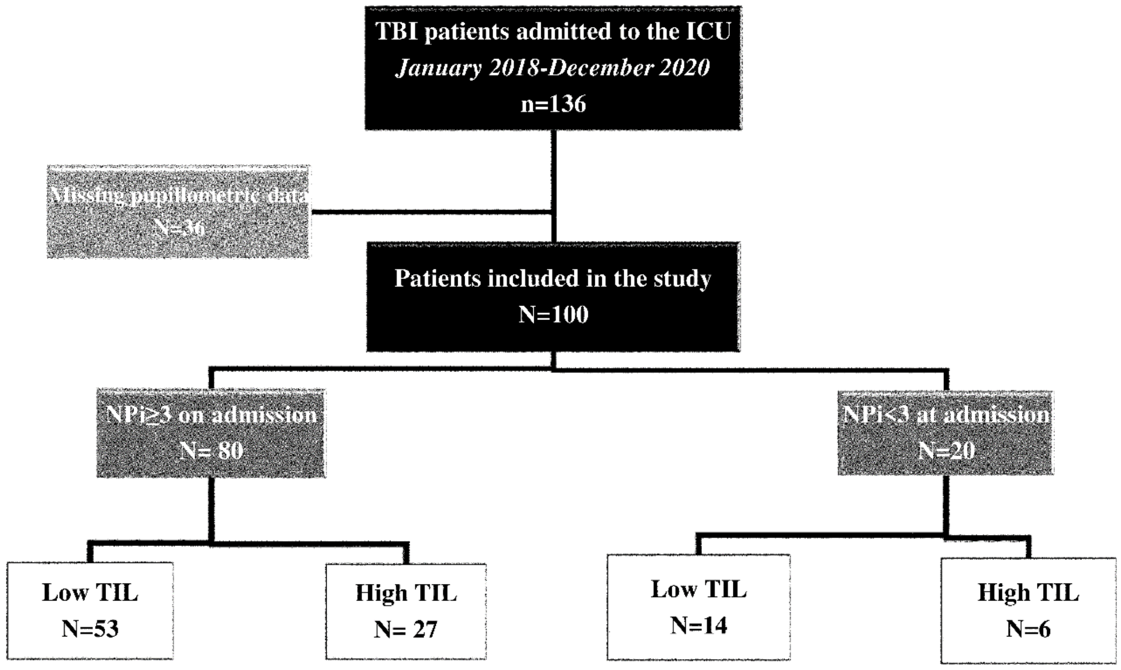

NPi = neurological pupil index; TIL = Therapeutic Intensity Level.

Supplemental Table S1. Characteristics of the study population, according to hospital mortality. Data are presented as count (%) or median (25th–75th percentiles).

	ALL (n = 100)	Non-Survivors (n = 29)	Survivors (n = 71)	*p value*
Age, (years)	48 [34–69]	67 [43–75]	43 [32–62]	0.002
Male Gender, n (%)	73 (73)	19 (66)	54 (76)	0.32
Mechanism of Trauma n (%)				0.17
Fall	64 (64)	23 (79)	41 (58)	
Car accident	24 (24)	4 (14)	20 (28)	
Aggression	9 (9)	1 (3)	8 (11)	
Other	3 (3)	1 (3)	2 (3)	
Polytrauma, n (%)	38 (38)	8 (28)	30 (42)	0.18
Glasgow Coma Scale GCS on admission	11 [6–15]	6 [3–12]	13 [7–15]	<0.001
SOFA Score on admission	6 [2–8]	8 [7–12]	4 [1–7]	<0.001
Marshall Score	5 [2–5]	5 [4–5]	4 [2–5]	0.06
Neurological Pupil Index				
Worst NPI at admission	4.2 [3.2–4.5]	3.6 [1.1–4.4]	4.3 [3.6–4.6]	0.01
Average NPI at admission	4.3 [3.4–4.7]	3.7 [2.1–4.4]	4.4 [3.8–4.7]	0.08
NPI < 3, n (%)	20 (20)	9 (31)	11 (15)	0.1
Worst size (max) on admission, mm	3.1 [2.4–4.2]	2.9 [2.4–4.1]	3.1 [2.4–4.2]	0.93
Worst CH (min) on admission, %	16 [9–26]	14 [1.5–20]	16 [9–27]	0.09
**Comorbidities**				
COPD/Asthma, n (%)	8 (8)	3 (10)	5 (7)	0.69
Chronic kidney disease, n (%)	7 (7)	4 (14)	3 (4)	0.19
Cirrhosis, n (%)	5 (5)	5 (17)	0	0.002
Heart disease, n (%)	24 (24)	12 (41)	12 (17)	0.01
Immunosuppression, n (%)	1 (1)	1 (3)	0	0.29
Previous neuro pathology, n (%)	19 (19)	9 (31)	10 (14)	0.09
Cancer, n (%)	5 (5)	3 (10)	2 (3)	0.14
Diabetes, n (%)	12 (12)	4 (14)	8 (11)	0.74
AHT, n (%)	25 (25)	14 (48)	11 (16)	0.001
Cataract, n (%)	3 (3)	1 (3)	2 (3)	1
Glaucoma, n (%)	1 (1)	0	1 (1)	1
Seizures, n (%)	5 (5)	1 (3)	4 (6)	1
Alcohol, n (%)	40 (40)	12 (41)	28 (39)	1
Smoking, n (%)	22 (22)	3 (10)	19 (27)	0.11
**ICU Therapies**				
Therapy Intensity Level (TIL-Basic)	2 [1–4]	1 [1–2]	4 [2–4]	<0.001
Sedative drugs, n (%)	68 (68)	28 (97)	40 (56)	<0.001
Analgesic drugs, n (%)	81 (81)	29 (100)	52 (73)	0.001

Vasopressor drugs, n (%)	56 (56)	24 (83)	32 (45)	<0.001
Inotropes drugs, n (%)	2 (2)	2 (7)	0	0.08
Mechanical ventilation, n (%)	69 (69)	27 (93)	42 (59)	0.002
ECMO, n (%)	1 (1)	1 (3)	0	0.29
ICP Monitoring, n (%)	45 (45)	18 (62)	27 (38)	0.04
Osmotic drugs, n (%)	46 (46)	20 (69)	26 (37)	0.004
Decompressive craniectomy, n (%)	29 (29)	14 (48)	15 (21)	0.009
Barbituric, n (%)	17 (17)	8 (28)	9 (13)	0.08
Hypothermia, n (%)	6 (6)	4 (14)	2 (3)	0.06
**Outcome Variables**				
ICU stay, days	6 [3–17]	9 [3–21]	5 [3–14]	0.18
Hospital stay, days	16 [8–43]	11 [4–24]	19 [10–48]	0.01
IMPACT Mortality, %	34 [15–50]	49 [41–63]	20 [9–37]	<0.001
IMPACT Unfavourable Outcome, %	50 [24–71]	71 [58–84]	34 [17–53]	<0.001

UO = Unfavorable Outcome; FO = Favorable Outcome; NPI = Neurological Pupil Index; SOFA = Sequential Organ Failure Assessment; CT = Computed Tomography; CH = Change (percentage of constriction); COPD = Chronic Obstructive Pulmonary Disease; AHT = Arterial Hypertension; ECMO = Extracorporeal Membrane Oxygenation; ICP = Intracranial Pressure; ICU = Intensive Care Unit; IMPACT = International Mission for Prognosis and Analysis of Clinical Trials in TBI.

Supplemental Table S2. ROC Curves according to prediction of hospital mortality

	AUC [IC 95%]	*p value*
Marshall score	0.67 [0.53–0.81]	0.03
TIL	0.62 [0.47–0.76]	0.15
TBI-IMPACT	0.85 [0.76–0.95]	<0.001
GCS on admission	0.72 [0.62–0.82]	0.001
Worst NPI both	0.66 [0.54–0.78]	0.013
Average-NPI	0.67 [0.55–0.79]	0.008
Worst Size both	0.56 [0.41–0.71]	0.47
Worst CH both	0.61 [0.49–0.73]	0.096

UO = Unfavourable Outcome; FO = Favourable Outcome; NPI = Neurological Pupil Index; CT = Computed Tomography; CH = Change (percentage of constriction); GCS = Glasgow Coma Scale; IMPACT = International Mission for Prognosis and Analysis of Clinical Trials in TBI; TIL = Therapy Intensity Level.

Supplemental Table S3. Characteristics of the study population, according to TIL-Basic. Data are presented as count (%) or median (25th–75th percentiles).

	**ALL (n = 100)**	**TIL 4 (n = 33)**	**TIL 1–3 (n = 67)**	***p value***
Age, (years)	48 [34–69]	42 [34–61]	49 [32–74]	0.26
Male Gender, n (%)	73 (73)	22 (67)	51 (76)	0.35
Mechanism of Trauma, n (%)				0.18
Fall	64 (64)	24 (73)	40 (60)	
Car accident	24 (24)	6 (18)	18 (27)	
Aggression	9 (9)	1 (3)	8 (12)	
Other	3 (3)	2 (6)	1 (2)	
Polytrauma, n (%)	38 (38)	12 (36)	26 (39)	0.83
Glasgow Coma Scale GCS on admission	11 [6–15]	4 [3–9]	7 [4–10]	<0.001
SOFA Score	6 [2–8]	8 [7–10]	7 [5–10]	<0.001
Marshall Score	5 [2–5]	5 [5–5]	3 [2–5]	0.002
Neurological Pupil Index				
Worst NPI on admission	4.2 [3.2–4.5]	3,8 [2.2–4.4]	4 [2.8–4.4]	0.05
Average NPI on admission	4.3 [3.4–4.6]	3,9 [2.2–4.4]	4.25 [3.2–4.5]	0.04
NPI < 3, n (%)	20 (20)	6 (18)	14 (21)	0.79
Worst size (max) on admission, mm	3.1 [2.4–4.2]	2.8 [2.3–4]	2.9 [2.3–3.9]	0.03
Worst CH (min) on admission, %	16 [8.5–25.5]	11 [0–14]	14 [6–17]	0.002
**Comorbidities**				
COPD/Asthma, n (%)	8 (8)	2 (6)	6 (9)	0.72
Chronic kidney disease, n (%)	7 (7)	2 (6)	5 (8)	1
Cirrhosis, n (%)	5 (5)	3 (9)	2 (3)	0.33
Heart disease, n (%)	24 (24)	7 (21)	17 (25)	0.81
Immunosuppression, n (%)	1 (1)	0	1 (2)	1
Previous neurological disease, n (%)	19 (19)	3 (9)	16 (24)	0.11
Cancer, n (%)	5 (5)	1(3)	4 (6)	0.66
Diabetes, n (%)	12 (12)	2 (6)	10 (15)	0.33
HTA, n (%)	25 (25)	6 (18)	19 (28)	0.33
Cataract, n (%)	3 (3)	0 (0)	3 (5)	0.55
Glaucoma, n (%)	1 (1)	0 (0)	1(1,5)	1
Seizures, n (%)	5 (5)	2 (6)	3 (4,5)	1
Alcohol, n (%)	40 (40)	15 (46)	25 (37)	0.52
Smoking, n (%)	22 (22)	7 (21)	15 (22)	1
**ICU Therapies**				
Sedative drugs, n (%)	68 (68)	33 (100)	35 (52)	<0.001
Analgesic drugs, n (%)	81 (81)	32 (97)	49 (73)	0.005
Vasopressor drugs, n (%)	56 (56)	31 (94)	25 (37)	<0.001

Inotropes drugs, n (%)	2 (2)	2 (6)	0 (0)	0.11
Mechanical ventilation, n (%)	69 (69)	33 (100)	36 (54)	<0.001
ECMO, n (%)	1 (1)	1 (3)	0 (0)	0.33
ICP Monitoring, n (%)	45 (45)	32 (97)	13 (19)	<0.001
Osmotic drugs, n (%)	46 (46)	32 (97)	14 (21)	<0.001
Decompressive craniectomy, n (%)	29 (29)	29 (88)	0 (0)	<0.001
Barbituric, n (%)	17 (17)	17 (52)	0 (0)	<0.001
Hypothermia, n (%)	6 (6)	6 (18)	0 (0)	<0.001
**Outcome Variables**				
ICU stay, days	6 [3–17]	2 [3–7]	12 [4–23]	<0.001
Hospital stay, days	16 [8–43]	15 [8–43]	22 [9–42]	0.08
ICU death, n (%)	27 (27)	14 (42)	13 (19)	0.02
Hospital death, n (%)	29 (29)	15 (46)	14 (21)	0.02
IMPACT Mortality, %	34 [15–50]	34 [27–55]	21 [9–49]	0.16
IMPACT Unfavourable Outcome, %	50 [24–72]	50 [40–71]	38 [18–71]	0.31
UO = GOS 1–3, n (%)	49 (49)	25 (76)	24 (36)	<0.001
Glasgow Outcome Scale	4 [1–4]	3 [1–4]	4 [3–4]	<0.001

UO = Unfavourable Outcome; FO = Favourable Outcome; NPI = Neurological Pupil Index; SOFA = Sequential Organ Failure Assessment; CT = Computed Tomography; CH = Change (percentage of constriction); COPD = Chronic Obstructive Pulmonary Disease; AHT = Arterial Hypertension; ECMO = Extracorporeal Membrane Oxygenation; ICP = Intracranial Pressure; ICU = Intensive Care Unit; IMPACT = International Mission for Prognosis and Analysis of Clinical Trials in TBI.

Supplemental Table S4. ROC Curves according to the prediction of high TIL during ICU stay.

**TIL 4 vs TIL 1–3**	**AUC [IC 95%]**	***p value***
Marshall CT score	0.72 [0.58–0.87]	0.004
TBI-IMPACT Mortality	0.61 [0.46–0.76]	0.16
TBI-IMPACT UO	0.58 [0.43–0.73]	0.31
GCS	0.73 [0.63–0.84]	<0.001
Worst NPI both	0.62 [0.51–0.73]	0.05
Average-NPI	0.62 [0.51–0.74]	0.04
Worst Size both	0.46 [0.31–0.61]	0.59
Worst CH both	0.69 [0.58–0.8]	0.002

TIL = Therapy Intensity Level; UO = Unfavourable Outcome; NPI = Neurological Pupil Index; CT = Computed Tomography; CH = Change (percentage of constriction); GCS = Glasgow Coma Scale; IMPACT = International Mission for Prognosis and Analysis of Clinical Trials in TBI.
